# From screening to enrollment drop-offs: perspectives of minoritized participants in Indiana

**DOI:** 10.3389/fpubh.2026.1858056

**Published:** 2026-08-19

**Authors:** Sylk Sotto-Santiago, Cynthia Brown, Brownsyne Tucker Edmonds, Cynthia Ziwawo, Molly Frank, Brenda Hudson, Sarah E. Wiehe

**Affiliations:** 1 Department of Medicine, University of Pittsburgh School of Medicine, Pittsburgh, PA, United States; 2 Department of Medicine, Indiana University School of Medicine, Indianapolis, IN, United States; 3 Indiana Clinical and Translational Sciences Institute, Indianapolis, IN, United States; 4 Department of Obstetrics and Gynecology, Indianapolis, IN, United States; 5 Department of Pediatrics, Indianapolis, IN, United States

**Keywords:** clinical trials, COVID-19, diversity, public health, recruitment, vaccine

## Abstract

Vaccine uptake among Black and Latine populations remains persistently lower than among white populations, yet drop-offs between screening and enrollment in clinical trials is underexamined. The current sociopolitical moment, marked by “DEI” rollbacks, disruptions of federally funded health research, and sensitivity to health equity language, heightens the urgency of this work and threatens infrastructure supporting equitable recruitment. This qualitative study investigated non-enrollment among individuals positively screened for a vaccine trial at Indiana University School of Medicine and the Indiana Clinical and Translational Sciences Institute. Seven themes emerged: institutional reputation; communication and awareness; healthcare professionals’ role; public and community engagement; recruitment practices; research participant motivation; and barriers to enrollment. From a critical lens, the findings of this study make clear that closing the gap between interest and enrollment in clinical trials requires more than improved recruitment messaging but structural transformation within every step.

## Introduction

In 2020, the SARS-COV2-19 (COVID-19) pandemic spread rapidly throughout the world and was associated with significant morbidity and mortality that affected historically marginalized communities disproportionately. The US government appropriated billions of dollars in research funding to support Operation Warp Speed to accelerate the development of vaccines against COVID-19, including more than $1billion for the development of AstraZeneca’s viral-vector vaccine AZD1222 (1). Inclusion of diverse populations in clinical trials was highly encouraged by the U. S. Food and Drug Administration (FDA) to ensure both safety and efficacy of vaccine candidates in marginalized populations (2).

At national and international levels, the lack of racial and ethnic representation in clinical trials precludes the generalizability and effectiveness of applying clinical findings to these populations (3). The importance of diverse recruitment into clinical trials cannot be understated and it is not limited to a pandemic. The equity concerns implicated by health disparities are important to highlight along with health inequities in our systems. Ensuring that historically marginalized communities have access to clinical trials may help improve disparities in health outcomes and promote just distribution of health resources (3).

Barriers to enrollment in clinical trials of vaccines for historically marginalized populations are varied and include general vaccine hesitancy, mistrust and distrust in biomedical and health-related research, health literacy, among others. Identification of specific strategies to overcome these barriers are important to improving enrollment in clinical trials, vaccine acceptance, and ultimately health equity in this country. Moreover, the 2025–2026 sociopolitical moment has heightened urgency during acute policy crisis. The current environment has produced two additional challenges in our efforts: rollback and decimation of health research funding, and sensitivity to health equity research language.

### The vaccine trial

Indiana University participated as a site in a Phase 3, double-blind, placebo-controlled vaccine trial conducted during 2020–2021, with follow-up consistent with standard Phase 3 vaccine trial protocols. The trial was conducted at 88 sites across the U. S. Chile, and Peru. Sites were chosen to participate in the trial based upon a variety of factors including access to a population of individuals at high risk of contracting COVID-19, appropriate research infrastructure and support, and the prevalence of circulating virus in the community. The FDA strongly encouraged enrollment of individuals from populations most affected by COVID-19 including racial and ethnic minorities (2). Although no target enrollment of minoritized groups was set by the FDA, the trial developed strategies to encourage recruitment of historically marginalized groups with a target enrollment of non-white individuals between 25–30%. Primary data from this trial has been published confirming vaccine safety and efficacy. Overall, 22.4% of the total population was of Hispanic/Latine ethnicity and 8.3% were Black/African American race with 21% being non-white race or ethnic group (1).

### All IN for health, Indiana clinical and translational science institute

Indiana University School of Medicine was chosen as a site for participation in the trial upon completion of a site feasibility assessment and was the only participating site for this trial in Indiana. Given the scope and timeline of the trial, recruitment goals were ambitious with a desired recruitment of 1,500 participants over a period of 8–12 weeks. To maximize recruitment at IU, the local study team engaged the assistance of the Indiana Clinical and Translational Science Institution (I-CTSI) Recruitment Services and more specifically All IN for Health. All IN for Health is an I-CTSI program dedicated to engaging the public in understanding the role and value of research towards improving the health of Indiana residents. The program strives to raise the voices of the public to identify research priorities, provide an opportunity to hear about research studies, and get connected to researchers, research results and findings. In what follows we present the screening and enrollment processes.

### Conceptual framework

Critical theories strive to understand, critique, and help overcome the social structures through which people are impacted, dominated, and oppressed. Several disciplines have incorporated this lens, including most recently the areas of critical public health and critical health studies. Both fields work to improve the conceptualization of race, ethnicity, and social and structural determinants’ effects on health. Through interdisciplinary awareness, they examine the conventions that intentionally and unintentionally reinforce health disparities and inequities. They also consider health systems, research opportunities, strategies, and policies that address access, perception, attitudes, and behaviors (4). Specifically, the public health critical race methodology considers contemporary patterns of racial relations, knowledge, production, conceptualization and action as potential dimension in this study (4).

We highlight that central to these critical frameworks is an explicit attention to power specifically, how power operates within research systems, healthcare institutions, and society to determine who participates in knowledge production, whose experiences are centered, and whose bodies bear the burden of both disease and scientific inquiry. Critical theories, including critical race theory interrogate how race functions not merely as a demographic variable but as a social and structural category produced through historical and ongoing relations of power. Within clinical research contexts, this manifests in who is recruited and how, who is deemed eligible, who can absorb the logistical demands of participation, and which populations have historically been harmed by or excluded from health research. The screening-to-enrollment gap documented in this study reflects the differential distribution of structural burdens and resources along racialized lines. Our analysis is therefore oriented toward understanding structural conditions and their relationship to power, not toward locating deficits within individuals or communities.

We view this present analysis as an opportunity to highlight the important contributions that racial and ethnic minoritized groups bring to the study of health disparities and to examine participation in clinical trials as means to challenge recruitment norms, question what has been defined as a problem, and break down assumptions.

## Methods

### Background: vaccine trial recruitment

Recruitment for the registry was conducted through a multi-channel media campaign coordinated by All IN for Health and the Indiana University School of Medicine study team. The campaign included local television appearances by the vaccine study principal investigator (CB), radio announcements, and social media outreach targeting Indiana residents. Interested individuals were directed to the All IN for Health online volunteer registry, where they completed an initial interest form. Those registered were subsequently invited via email to complete a formal screening questionnaire to assess initial eligibility for the trial. More than 3,000 individuals who volunteered for the registry were invited to complete a screening questionnaire to determine initial eligibility for trial participation. Of those invited to complete screening 1,002 completed the questionnaire. The race/ethnicity of individuals completing this questionnaire is shown in Table 1. Further review of the screening questionnaire determined initial eligibility, and these individuals would be offered a visit in person to complete informed consent and randomization in the trial. As shown in Table 1, the percent of patients randomized who were ultimately enrolled was lowest for Black and Indigenous American although the latter had very small numbers.

**Table 1:** Vaccine trial screening process data.

Race/ethnicity	Screen		1st Eligible/invited		2nd Eligible/randomized		Percent of initial screen	Percent of initially eligible
White (non-Hispanic)	1,002	71%	752	76%	413	78%	55%	60%
Black, African- American	101	7%	66	7%	24	5%	24%	36%
Asian/Asian American	38	3%	31	3%	15	3%	39%	48%
Hispanic/Latine	149	11%	114	11%	61	12%	41%	53%

As shown in Table 1, the percentage of individuals who were ultimately enrolled following randomization was lowest among Black/African American participants; American Indian/Alaska Native individuals are not shown in Table 1 due to small cell sizes (*n* < 5) but are addressed narratively where relevant.

This research explores the reasons for this drop-off between eligibility to enrollment through semi structured interview of these individuals to explore their experiences as part of the process and perspectives about clinical trials and determine their hesitation to participate. Participants who completed the qualitative interview were offered a $25 gift card as compensation for their time. No additional incentives were offered for registry enrollment or screening participation in the original trial.

We used purposive sampling of individuals that were successfully screened but did not enroll in this study, inviting one hundred and two individuals to be part of this study. The interviews covered the following topic domains:

1. Communication and awareness: e.g., “How did you first hear about this clinical trial?” and “Was the information you received clear and sufficient for you to make a decision?”
2. Trust and institutional reputation: e.g., “What is your perception of Indiana University School of Medicine as a research institution?” and “To what degree did trust in the institution influence your decision?”
3. Healthcare provider involvement: e.g., “Did you discuss your potential participation in this trial with any of your healthcare providers?”
4. Motivation and willingness to participate: e.g., “What motivated you to sign up for the registry in the first place?” and “What factors, if any, would make you more likely to participate in future clinical trials?”
5. Impact of the pandemic: e.g., “How did the COVID-19 pandemic itself affect your decision to participate or not participate?”
6. Barriers to enrollment: e.g., “What were the primary reasons you did not complete the enrollment process?” covering logistics, the informed consent process, competing demands, and scheduling.
7. Academic medical center mission and community responsibility: e.g., “What do you think is the responsibility of academic medical centers to communities like yours when conducting research?”

The semi-structured interview guide informed the initial analytic categories, which were then refined through the constant comparative method; final themes reflect both deductive (guide-informed) and inductive (emergent) dimensions of participants’ responses.

Data collection continued until the research team determined that saturation had been approached, defined in this study as the point at which no substantively new themes or categories emerged across interviews. Given the sample size (*n* = 14), the team applied a conservative interpretation of saturation, recognizing constrains by both the self-selected nature of respondents and the demographic composition. Saturation was assessed iteratively during analysis through ongoing team discussion (SS, MF, CZ) after each round of coding. While the team is confident that the themes identified reflect meaningful patterns, we acknowledge that the saturation standards reflect conceptual consistency within the sample rather than generalizability among broader populations of interest. In addition, the clinical trial itself was hampered by truly engaging with communities due to pandemic restrictions and the urgency for recruitment.

Data was analyzed using the constant comparative method. Researchers (SS, MF, CZ) assessed responses to interview questions, categorized answers according to the perspectives expressed in the interviews, re- examined and integrated categories (SS, MF, CZ). The advantage of this method was the opportunity to explore themes in greater depth through comparison. Moreover, throughout design, conceptualization, data collection, and analysis, the coding team (SS, MF, CZ) held debrief discussions after each phase, detailing assumptions about participants’ motivations and question our own framing rather than the team’s “hypothesis” or expectations, particularly with respect to race and ethnicity. When disagreements arose about how to categorize a response, the team returned to the transcript and discussed until consensus was reached. This iterative process was intended to guard against reproducing deficit-based narratives about minoritized participants, which is consistent with the public health critical race methodology guiding this work.

### Research team positionality

Our research team includes individuals across multiple racial, ethnic, and disciplinary backgrounds, including members who identify as Black, Latine, and white, and who hold clinical, public health, health equity, research ethics, and community-engagement expertise. Several members bring lived experience navigating healthcare and research systems as members of minoritized communities, while others bring institutional vantage points as clinician-researchers within the academic medical center that served as the trial’s recruiting site. We recognize that our positions within these systems, either as insiders to the recruiting institution, others as members of communities historically underserved or excluded by research, shape both the questions we asked and the interpretations we offer. Rather than treating these differences in relation to race, power, and institutional position as a limitation to be resolved, we treated them as an analytic and asset-based skill throughout the study.

## Results

We invited 102 persons to participate in survey through email (allowed due to IRB approved consent in the All IN for Health registry), 27 people responded that they would like to participate, 1 person only partially completed the information, 74 people never responded after 3 attempts to reach them; only 14 people were able to complete the interview. The proportion of individuals who participated versus were eligible is shown in Table 2.

**Table 2:** Participants.

Final cohort interviewed						
Race	Ethnicity	Age eange	Offered participation	Female	Male	Total
White	Hispanic/Latine	18–40	23	5		5
		41–64	18	1	1	2
		65 and older	2		1	1
		No Age Info	1			0
Black/African American	Non-Hispanic/Latine	18–40	12			0
		41–64	20	1	2	3
		65 and older	4		1	1
		No Age Info	1			0
More than 1 Race or Other Race	Non-Hispanic/Latine	18–40	3			0
		41–64	2	1	1	2
Native American/Alaskan Native	Hispanic/Latine	18–40	1			0
Native American/Alaskan Native	Non-Hispanic/Latine	18–40	0			0
		41–64	4			0
Asian	Non-Hispanic/Latine	18–40	5			0
		41–64	2			0
		65 and older	2			0
		Total	101	8	6	14

Outcomes of this study were categorized through seven themes: academic medicine mission and institutional reputation; communication and clinical trial awareness campaign; healthcare professionals; public and community engagement; recruitment practices; motivation and willingness to participate in research; and barriers to enrollment. Themes are discussed by frequency of occurrence: (see Table 3)

**Table 3:** Themes, definitions, and frequency of occurrence (*N* = 14).

Theme	Definition	*n* (of 14)	%
Barriers to enrollment	Logistical, structural, and process-based obstacles that prevented screened individuals from completing enrollment, including informed consent complexity, geographic distance, scheduling burden, and competing life circumstances.	12	86%
Motivation and willingness to participate	Intrinsic and extrinsic factors driving initial interest in trial participation, including altruism, personal/family health history, civic responsibility, and desire to contribute to science.	12	86%
Academic medicine mission & institutional reputation	Participants’ perceptions of the sponsoring academic medical center’s credibility, trustworthiness, and stated commitment to community health.	11	79%
Recruitment practices	Participants’ evaluations of the practical dimensions of the recruitment process, including staff interactions, clarity of information, and logistical support.	10	71%
Communication and clinical trial awareness	Awareness of and reactions to the media campaign promoting the trial, and perceived adequacy of pre-enrollment information.	9	64%
Public and community engagement	Perspectives on the role of community organizations, faith institutions, and local leaders as partners in research dissemination and trust-building.	8	57%
Healthcare professionals’ role	Provider involvement (or absence thereof) in participants’ decision-making about trial participation. Low frequency but high analytical significance given existing literature.	1	7%

Frequency counts reflect the number of participants who raised each theme spontaneously or in response to direct questioning. Intensity was assessed qualitatively through team discussion during iterative coding.

### Logistical barriers to enrollment

Barriers clustered into three primary categories. First, the informed consent process. Participants described receiving a lengthy written packet enumerating potential side effects without accompanying explanation or guided discussion. Several participants appreciated the transparency, with most finding the document daunting. The delivery of a complex informed consent document as a document-delivery event rather than a supported conversation appears to have functioned as a barrier in multiple cases. Second, geographics. Distance to the Indianapolis trial site, the volume of required follow-up visits, and time commitment were cited consistently. Distance to the Indianapolis trial site emerged as a barrier in several overlapping ways: participants described the burden of arranging transportation for repeated visits, coordinating time away from work or childcare responsibilities to accommodate visit schedules, and, for participants living in more rural areas of the state, the cumulative time of travel. These logistical demands compounded across the multiple required follow-up visits, disproportionately burdening participants without flexible work schedules or reliable transportation. Lastly, external competing factors including contracting COVID-19 before enrollment or obtaining access to another vaccine through alternative channels resulted in 2 additional drop-offs that could not have been anticipated at the recruitment stage.

### Motivation and willingness to participate

Participants described genuine motivations for their initial interest, including personal and family health histories, desire to contribute to scientific knowledge, and civic responsibility during a pandemic that had disproportionately harmed communities of color. These findings are consistent with existing literature documenting research altruism as a primary driver of trial interest among minoritized populations (5).

### Academic medicine Mission and institutional reputation

First, participants recognized the source of the recruitment as a large academic medical center with strong reputation in the state: “*It’s been one that has been trusted for quite some time. Um, I know they have a really good long reputation as far as, you know, it’s a school of medicine. This is where people are going to learn about medicine, to learn how to take care of people, to learn how to help a human and a human life, you know, to make sure that they stay healthy.”* And *“The school itself is known for innovation, integrity, um, just being go-getters when it comes to medical research, cancer research. Just, um, so many wonderful things I can say about IU. Um, and it’s certainly a hospital that has an astounding reputation and a hospital that I trust even with the reputation put aside. Every time I have a doctor’s appointment with either a GP or a specialist, I’ve always had a really good relationship with these. I trust them. Yeah, with the physicians.”* Thus, the source of the organization or institution matters when it comes to trusting the source of the clinical trial.

### Recruitment practices

Participants were largely positive about the practical dimensions of the recruitment process. Recruitment is more than advertising strategies; it requires recruitment and study teams to identify potential participants, clearly discuss all aspects of the trial, ensure full comprehension of requirements and expectations, and most importantly, establish trust with potential participants.

Participants overwhelmingly expressed their satisfaction with the research staff interactions and pointed to three areas that were critical for their enrollment: ease of potential participation, health research literacy and comprehension of safety concerns, and logistics. Participants were pleased: *“The ability to indicate my interest in participating and the easiness with which one could express their interest [was good]. “I think more information at that time would have been helpful, but there was enough to excite someone about getting involved.”* Logistics were also clearly explained: *“[they were] helpful like they were doing everything safe and yeah, they send me the parking and how to get to the clinic and all of that.*

### Communication and clinical trial awareness

The media campaign promoting the trial was perceived as effective. The principal investigator (CB) appeared across television, radio, and social media. One participant described being motivated by messaging that framed trial participants as civic contributors *“the first batch of people”* to try the vaccine for humanity. However, participants also noted that promotional materials did not adequately prepare them for the complexity of the enrollment decision. One participant reported that information *was “enough to excite someone about getting involved”* but that more substantive detail earlier would have been helpful.

### Public and community engagement

Participants expressed great interest for more sustained institutional-community partnerships. Recommendations centered on models in which community organizations, faith communities, and local leaders serve as genuine research stakeholders, not just as dissemination channels. Their responses reflect a principle well-established in the community-engaged research literature: trust is built through reciprocal relationship over time, not through outreach concentrated in a single recruitment phase (6).

### Healthcare professionals’ role

Although low in absolute frequency, this finding is reported because of its strong alignment with existing literature identifying provider involvement as a critical structural factor in research participation, its near-complete absence in our sample warrants attention. This finding is among the most practically significant in the study. Provider, especially primary care providers, are consistently identified in the literature as among the most powerful predictors of patient engagement in both health behaviors and research participation (7). For populations in which the patient-provider relationship is often the most trusted health information channel available, failure to integrate providers into the recruitment pathway can contribute to enrollment drop-off.

## Discussion

Our study examined an understudied “failure point” between screening and enrollment in a vaccine trial. Early interest is a great sign of potential enrollment, yet it does not translate into equal levels of final enrollment. Several findings contribute to our current realities. First, the near-total absence of healthcare provider involvement in participants’ enrollment decisions represents a structural gap in the US. Scholars have demonstrated that the extent of provider involvement is a key driver of vaccine uptake and health research participation (8). Second, the informed consent, meant for regulatory purposes and medical transparency, continues to lack a structured dialogue, a challenge and reminder in research ethics. Third, participation impacted by key aspects of structural drivers of health, such as employment, geographic location, etc. proved once again that the populations with the greatest burden are the least able to absorb the time commitment and costs of clinical trial participation.

Our study adds to existing evidence of challenges and barriers to health research participation. However, from a criticality lens, we continue to identify patterns of enrollment attrition among Black/African American and Hispanic/Latine populations, which have nothing to do with willingness to participate. Moreover, rather than attributing these gaps to simply vaccine hesitancy, we can situate them within structural racism that determines who bears the greatest disease burden while simultaneously remaining least positioned to participate in health research. As mentioned, the near-total absence of healthcare provider involvement, the “race-ethnicity neutral design” of informed consent processes, and the structural drivers of health represent broadly known shortcomings of our research infrastructures.

The “failure point” between screening and enrollment is not a point in a recruitment timeline, it is a clear patterned outcome produced by structural conditions. In many ways, it reflects normalized inequities in how healthcare is organized and delivered across racialized lines. The COVID-19 pandemic created a moment when vaccine trial enrollment was urgently needed across all populations, yet this proved to be insufficient in overcoming structural barriers. Shared interest alone does not dismantle the inequities in our health system and research infrastructures.

Also, we take this opportunity to reaffirm trust and trustworthiness as two of the greatest drivers in health research (6, 9). Therefore, we join others in considering trust as a social determinant of health. Trust influences individual experiences and health outcomes and should be considered in the public discourse. The more distrust in the patient-provider relationship, the more health disparities exist. Trust enables acceptance of evolving science, allows acceptance of public health measures, and mitigates health consequences of social disparities (8, 10, 11).

Despite this study’s focus on understanding enrollment attrition among Black/African American and Hispanic/Latine populations, our final interview sample remained disproportionately, reflecting both the demographic composition of the originally screened population and differential response rates to the interview invitation. As such, the perspectives of Black/African American and Hispanic/Latine individuals who screened positive but did not enroll remain incomplete. Never should quotes represent these groups’ experiences overall.

Future scholarship must center the structural and social determinants of health through an explicit anti-racist framework, ensuring that minoritized communities are recognized not as problems to be recruited around, but as essential partners in the redesign of equitable research and health systems. We realize that this simple suggestion, has incredible weight today.

## Conclusion

The findings of this study make clear that closing the gap between interest and enrollment in clinical trials requires more than improved recruitment messaging, it demands structural transformation. Patterns of attrition are not trivial; they are the predictable outcomes of research systems built without adequate attention to the communities they most need to serve. Addressing this will require sustained investment in provider-patient relationships that bridge communities into research and the removal of structural barriers that make participation inaccessible for those already navigating the greatest health burdens. Most critically, it will require a shift in how academic medical centers position historically minoritized communities not as hard-to-reach populations, but as co-architects of the research and experts in their own health. We acknowledge that calling for an explicit framework in health research carries significant weight in the current political and institutional climate. Yet, the evidence compels us not to forget this. Equity in health research participation is not a secondary concern it is foundational to the integrity and impact of public health.

## Data Availability

The raw data supporting the conclusions of this article will be made available by the authors, without undue reservation.
